# Treatment of Ringworm of the Scalp by Means of the X-Rays

**Published:** 1908-08-08

**Authors:** 


### Dermatology.

### TREATMENT OF RINGWORM OF THE SCALP BY MEANS OF THE X-RAYS.

It has for long been recognised that it is impossible to cure ringworm of the scalp by the application of parasiticides, owing to the impossibility of reaching the fungus in the deeper parts of the hair-follicle. The more rational treatment is to remove the hair upon and in which the fungus is growing. But if an attempt to do this is made by traction on the hair, the latter breaks off in the follicle, leaving the root and part of the fungus-invaded shaft behind. If, however, the ringworm patches can be inflamed by strong local applications, the infected hair-stumps will fall out, carrying with them the fungus, and leaving bald areas, upon which eventually the hair grows again. Many applications of this sort have been recommended from time to time, such as croton-oil, glacial acetic acid, and formalin. But these drugs are liable to produce too much inflammation, and without great care their application may result in scarring and permanent baldness. Other preparations employed are alcoholic solutions of iodine, ointments of nitrate of mercury, of sodium chloride, etc.; but all of these are uncertain in their action, and it is only in a small proportion of cases that they produce the desired inflammation of the ringworm patches, and consequent fall of hair over these areas.

Of recent years the *x*-rays have been employed as a means of treatment because it has been found that by their application the hair of the part exposed to the rays may be made to fall out, and, if applied with proper precautions, without any danger of permanent loss of hair. The *x*-rays have no antiparasitic action; they do not destroy the fungus; they merely cause the hair to fall. The fungus, however, comes away with the hair, and as there is an interval of some two months before the new hair begins to grow again, it has no chance of reinfecting the latter. The *x*-rays do not discriminate, but the whole of the hair, both diseased and sound, over the part exposed to their action, falls out.

If there are only a limited number of ringworm patches, each of these (and a portion of the sound hair around) may be treated separately. Care must be taken to protect the rest of the scalp from infection during the period that the diseased stumps are falling by the application of a parasiticide to the whole of the scalp. In very many cases, however, there are patches or isolated stumps all over the scalp, so that the whole of the scalp must be depilated.

By the introduction of means of accurate measurement it is now possible to give the whole amount of *x*-rays necessary to cause fall of the hair over any part exposed to their action in one dose. Indeed, the method by single-measured doses is the only safe one. It is a curious fact that the action of the rays is delayed for some time after the actual application, and the fall of the hair takes place from 14-18 days after the exposure. The operation is altogether without pain to the patient, and if the proper dose is given there is no inflammatory reaction of any sort, but merely fall of hair two weeks after the dose has been given. If too small a dose is given the hair will not fall. If too large a dose is given a subsequent erythema or dermatitis results, and permanent baldness is produced. But with modern methods of measurement this should never occur, and with ordinary precautions the treatment is quite free from any danger of permanent loss of hair.

The technique of the operation is as follows:—The hair having been cut short, the diseased areas are ringed round with a blue-pencil mark to indicate their position. If there are a few isolated patches these may be exposed singly. The *x*-ray tube is enclosed in a shield, opaque to the rays, except at an orifice upon one side. Upon this orifice are fixed lead-glass cylinders, or localisers, of different sizes, according to the dimensions of the area to be exposed. The length of the cylinders is such that the exposed area is seven inches from the source of the rays. The part of the head to be treated is then made to rest against the end of the glass cylinder, and a Sabouraud pastille is fixed midway between the source of the rays and the scalp. The tube is then put into action, and the exposure is continued until the pastille has changed from its normal bright green colour to a standard yellow tint. The dose sufficient to produce epilation has then been given, and within three weeks from the time of exposure the area treated will be quite bald. In two months more the hair will be growing again.

When the whole scalp has to be treated (as is most often the case) the operation is much less simple. The scalp is then mapped out into from eight to ten areas, each of about three to four inches in diameter, and each of these areas is treated separately. The very greatest care is needed to avoid any overlapping and consequent double dose at the contiguous parts of adjacent areas. The exposures are best given at one sitting, so that the lines marking the boundaries of the areas are not lost sight of. The average time for each exposure is fifteen to twenty minutes, so that (allowing for the time occupied in adjusting the apparatus for each dose) the whole treatment occupies from two and a half to three or four hours.

